# Impacts of Temperature on Primary Productivity and Respiration in Naturally Structured Macroalgal Assemblages

**DOI:** 10.1371/journal.pone.0074413

**Published:** 2013-09-13

**Authors:** Leigh W. Tait, David R. Schiel

**Affiliations:** Marine Ecology Research Group (MERG), School of Biological Sciences, University of Canterbury, Christchurch, New Zealand; University College Dublin, Ireland

## Abstract

Rising global temperatures caused by human-mediated change has already triggered significant responses in organismal physiology, distribution and ecosystem functioning. Although the effects of rising temperature on the physiology of individual organisms are well understood, the effect on community-wide processes has remained elusive. The fixation of carbon via primary productivity is an essential ecosystem function and any shifts in the balance of primary productivity and respiration could alter the carbon balance of ecosystems. Here we show through a series of tests that respiration of naturally structured algal assemblages in southern New Zealand greatly increases with rising temperature, with implications for net primary productivity (NPP). The NPP of *in situ* macroalgal assemblages was minimally affected by natural temperature variation, possibly through photo-acclimation or temperature acclimation responses, but respiration rates and compensating irradiance were negatively affected. However, laboratory experiments testing the impacts of rising temperature on several photosynthetic parameters showed a decline in NPP, increasing respiration rates and increasing compensating irradiance. The respiration Q_10_ of laboratory assemblages (the difference in metabolic rates over 10°C) averaged 2.9 compared to a Q_10_ of 2 often seen in other autotrophs. However, gross primary productivity (GPP) Q_10_ averaged 2, indicating that respiration was more severely affected by rising temperature. Furthermore, combined high irradiance and high temperature caused photoinhibition in the laboratory, and resulted in 50% lower NPP at high irradiance. Our study shows that communities may be more severely affected by rising global temperatures than would be expected by responses of individual species. In particular, enhanced respiration rates and rising compensation points have the potential to greatly affect the carbon balance of macroalgal assemblages through declines in sub-canopy NPP, the impacts of which may be exacerbated over longer time-scales and could result in declines in sub-canopy species richness and abundance.

## Introduction

Increasing greenhouse gas emissions are already causing rapid changes in the Earth’s climate [1] and by the end of this century, CO_2_ concentrations are expected to double or triple and global average sea-surface temperatures are projected to increase by 1.4-5.8°C. Although the ecological consequences of anthropogenic climate change are being observed [2-9], quantifying the full impacts on ecosystem functioning remains elusive.

It is known that many algal species have increased gross primary productivity (GPP) at higher temperatures [10,11], but net primary productivity (NPP) can be affected by different light environments and larger increases in respiration rates [12]. In natural communities, particularly on temperate shores dominated by stands of fucoid algae, there is layering of canopies and so a quite variable light environment from above the canopy down to the primary substratum [13,14]. Immersed macroalgal assemblages can have high primary productivity [15] and even during emersion there can be moderate rates of NPP [16-18]. Therefore, it is plausible that responses to increasing temperature will also have a variable relationship as single species are tested within their natural communities where light is filtered through assemblage canopy layers [19-21]. Sub-canopy components of assemblages are exposed to a low light environment, and may be susceptible to large rises in respiration rates with increasing temperature [22]. Furthermore, we have shown that laboratory-based studies provide a good baseline of production dynamics [23], but because of differences in the light environments between laboratory and *in situ* conditions a combination of techniques would be helpful in clarifying the responses of species and assemblages to changing temperature.

There have been numerous studies detailing the effects of rising temperature on the responses of individual plants and algal species [10,11,24], but little has been done to examine the effects of rising temperatures on assemblages or communities [7], particularly in natural settings. Although the species-specific responses to rising temperatures can uncover the mechanisms of thermal stress, they are unable to account for species interactions [25]. One of the few studies examining the consequences of climate change on macroalgal assemblages showed that thermal effects depended on the composition of canopy and understory species [26].

Variable responses of community components have the potential to either buffer or amplify the impacts of thermal stress. Metabolic processes such as primary productivity and respiration typically increase with temperature, known as the Q_10_ effect (the rate of change in processes over 10°C), but they potentially can become mismatched [12,27]. Metabolic processes are enhanced by rising temperature, and usually double with every 10°C rise in temperature (i.e., have a Q_10_ of 2), but can vary considerably among species and experimental conditions [24,28]. At very high light intensities, photoinhibition can be intensified with rising temperatures, resulting in a large decline in photosynthesis [22,29,30]. Furthermore, in seagrasses [11] and macroalgae [10], higher temperatures have an increasingly negative effect on photosynthesis at low, sub-saturating irradiance. Although both photosynthesis and respiration increase at progressively higher temperatures, respiration often increases at a faster rate [22,27,31], and primary productivity can be compromised at low light intensities [10,32]. Therefore, responses of assemblages to temperature could be positive or negative depending on light conditions and assemblage structure. In the kelp *Saccharinalatissima* (formerly *Laminaria saccharina* (L.) Lamouroux), for example, light-use efficiency (*α*) declines with increasing incubation temperature [10], leading to an increase in the compensation point (or *E*_*c*_, the light intensity at which photosynthesis and respiration are equal) and thus a reduction in net photosynthesis.

The interaction between low light and rising temperatures may negatively affect net primary productivity (NPP) in macrophyte communities. This is increasingly relevant in coastal marine areas where widespread sedimentation and eutrophication occur because of changes in human land use [33,34]. On a local scale within algal assemblages, the level of irradiance reaching the understory is often only a small fraction of that reaching the overlying canopy [19,20]. Communities are expected to have a higher respiration rate than any photosynthetic element within that community because most phyto-elements of the assemblage are not light-saturated, even at the highest light intensities [21], and species or photosynthetic elements at lower canopy levels may be susceptible to even small changes in the light or temperature climate. The effects on real, multi-layered communities may, therefore, be quite different from those on species studied in isolation.

In this study we use laboratory and *in situ* photorespirometry to determine the effects of temperature on NPP of intertidal algal assemblages. We used field-based experiments to test the effects of natural variation in temperature and irradiance on NPP of fucoid-dominated assemblages over one year, thereby incorporating photo-acclimation and temperature acclimation responses due to seasonal changes in the irradiance and temperature environment. *In situ* assemblages were then modified to examine the impacts of the seasonal temperature and irradiance gradient on the isolated community components of the canopy and sub-canopy alone. To clarify how the photophysiology of these assemblages responds to temperature changes, the laboratory portion of this study tested the acute effects of rising temperature on several photosynthetic parameters of the fucoid and its associated assemblage, which included the canopy, sub-canopy and basal components. We tested the hypotheses that a) rising temperature will have a greater negative impact on macroalgal assemblages than on single thalli, and b) respiration will be more severely affected than NPP in multi-layered assemblages.

## Methods

To understand how *in situ* macroalgal assemblages may be impacted by climate change, we tested the influence of natural seasonal temperature ranges on several photosynthetic parameters of assemblages varying in complexity. Although these methods have limitations due to the often confounded variation of responses of these variables to temperature, irradiance and acclimation of photosynthetic apparatus, they highlight some of the possible relationships between temperature and aspects of photophysiology in complex assemblages. To rectify some of these problems in the *in situ* part of this study, we used a complementary set of experiments under laboratory conditions to test the mechanisms and wider implications of results from field observations.

Three sets of experiments were done to test net primary productivity (NPP) and other photosynthetic parameters across light and temperature gradients: 1) experimental manipulations of assemblage layers within *in situ* communities across seasons; 2) laboratory-based measurements of NPP on single species; and 3) laboratory-based experiments of assemblages of multiple species, including canopy, sub-canopy and basal components.

### In situ photorespirometry

All experiments were done in assemblages with a dense canopy of the fucoid *Hormosira* *banksii* (Turner) Descaisne, which commonly dominates shores in southern New Zealand and southeastern Australia. Field experiments were done at Wairepo Reef, Kaikoura [35]. Assemblages were dominated by three main components: 1) the fucoid canopy species *H* *. banksii* (average percent cover, 88.25 ±7.6%; average dry weight, 70.6 ±20.4 g); 2) sub-canopy algae, including juveniles of the fucoids *Cystophora* *torulosa* (average percent cover, 16.5 ±5.4%; average dry weight, 15.1 ±4.4g) and *Carpophyllummaschalocarpum* (average percent cover, 4.75 ±4.3%; average dry weight, 15.8 ±6.2g), ephemeral algae such as *Champianovouzealandia* (average percent cover, 8.7 ±4.9%; average dry weight, 9.1 ±4.6g); and 3) the basal assemblage dominated by *Corallina* *officinalis* (average percent cover, 38.25 ±5.3%; average dry weight, 33.1 ±13.3g).

To record natural conditions to be used in conjunction with photorespirometry experiments at the field site, irradiance and temperature were logged in the mid-shore of Wairepo Reef using HOBO (Onset^©^) loggers; irradiance was cross-calibrated with a LiCor meter (LI-192 quantum sensor) to convert light measured by HOBO loggers into PAR irradiance in µmol m^-2^ s^-1^. Irradiance and temperature were recorded at 5 minute intervals between February 2008 and March 2010. Loggers were set facing upwards on the reef and fixed securely so that sensors could not change orientation. These were checked routinely to prevent fouling. Irradiance was also measured simultaneously above and below the canopy of *H* *. banksii* to determine the light reaching the sub-canopy. Irradiance above the canopy (ambient) was approximately 40 times higher than in the sub-canopy. Sub-canopy irradiance (i.e., on top of *Corallina* but below the canopy and sub-canopy layers) averaged just 18 µmol m^-2^ s^-1^ at an ambient irradiance of 500 µmol m^-2^ s^-1^, 28 µmol m^-2^ s^-1^ at an ambient irradiance of 1000 µmol m^-2^ s^-1^, and 50 µmol m^-2^ s^-1^ an at ambient irradiance of 2000 µmol m^-2^ s^-1^.

NPP of natural macroalgal assemblages was examined *in situ* using custom-built incubation chambers fixed to the substratum of the reef [23]. Chambers were designed to be secured around established assemblages of benthic intertidal algae without displacing them. They were made of a clear Perspex tube of height/volume 25 cm/12.3 l with a clear Perspex attachment plate and lid [23]. They were attached to the reef using a separate base plate to which the main chambers were bolted. The base plate was secured to the reef using four stainless steel bolts fixed into rawl plugs in the substratum. The Perspex tubing had an internal diameter of 25 cm (8mm thick) and covered a reef surface area of approximately 491 cm^2^. A submerged bilge pump was used to mix the chambers (as in the laboratory-based experiments) and stop the formation of boundary layers.

Incubations were done while the tide covered the chambers to ensure that the internal temperature remained stable and there was no formation of oxygen bubbles, and also to ensure that the light regime was as natural as possible. Temperature within the chambers was measured throughout experiments using HOBO (Onset Corporation ™) data loggers and irradiance was measured with a LiCor meter (LI-192 quantum sensor). The temperature loggers were placed on the inside of the chamber and irradiance was measured outside of the canopy, but beneath the 1cm thick lid.

Oxygen exchange was measured by removing water samples using 50 mL syringes. A Hach LDO probe (Model HQ40d) was used to test the dissolved oxygen (DO) concentration of the seawater within the syringe. Respiration measurements were taken by covering chambers to omit light or during night incubations. No significant differences in respiration were found between night incubations and those done in covered chambers during daytime. Invertebrates were removed from assemblages by hand, and by using a low pressure water pump to flush remaining invertebrates and sediments from the coralline turf. Natural variation in temperature was used to determine the effects of temperature on NPP and respiration across irradiance levels. Photosynthesis-irradiance (*P-E*) curves were generated from incubations at three temperature ranges: 8-12°C (winter), 13-17°C (autumn) and 18-22°C (summer). NPP was standardized by dry weight of algae (mg O_2_ gDW^-1^h^-1^) for analysis, but was presented by area (g O_2_ m^-2^h^-1^) for comparison with laboratory assemblages.

*In situ* NPP measurements were done during austral summer (December 2009), autumn (March 2010), and winter (June 2010). To test potentially variable responses of the sub-canopy parts of the assemblage in different light regimes, three canopy treatments were initiated. In a series of replicate plots, the canopy was removed, leaving the sub-canopy intact, and in others the sub-canopy was removed (including mid- and basal components), leaving the canopy intact. The three canopy treatments were intact assemblages (control), the canopy minus sub-canopy, and the sub-canopy minus the canopy (hereafter referred to as intact, canopy and sub-canopy). NPP was analyzed at irradiances of approximately 1000 μmol m^–2^ s^-1^ and 1900 μmol m^–2^ s^-1^ for three replicates of each treatment. Because irradiance data were based on natural variation of light (i.e., there was no shading of chambers to reach desired light intensities), data at around 1000 μmol m^–2^ s^-1^ and 1900 μmol m^–2^ s^-1^ were binned using data between 900-1100 μmol m^-2^ s^-1^ and 1800-2000 μmol m^–2^ s^-1^, respectively.

### Algal collection and acclimatization

Intact macroalgal assemblages were removed from the mid-intertidal zone of Wairepo Reef, for laboratory incubations during austral autumn (April-May) 2009. These assemblages were chiseled from the reef with the substratum attached (approximately 15 × 15 cm of substratum), then taken back to the laboratory where incubations were done. Algal collections were done under the collecting permit of the School of Biological Sciences, Canterbury University from New Zealand’s Ministry of Primary Industries.

Algae were kept in a re-circulating seawater system, at 10°C ±0.3 and a 12-hour light/dark cycle. Before experimentation all assemblages were given at least 48 hours (and not more than 56 hours) to acclimatize before experiments were initiated. The temperature range of the coastal water of southeastern New Zealand varies considerably throughout year, with the Kaikoura coast receiving annual sea-surface temperatures ranging from 9-18°C [36], but intertidal platforms regularly reach temperatures above 20°C during the summer months and below 7°C in the winter. Therefore, the experimental range tested was 10, 15, 20 and 25°C.

### Laboratory photorespirometry- Single thalli and assemblages

To clarify the impacts of temperature stress on the photophysiology of the fucoid assemblages we manipulated temperature in a series of complementary laboratory experiments. *In situ* tests of temperature variation necessarily confounded seasonal acclimation and daily light variation with temperature and light differences. Laboratory incubations were used to identify the acute effects of rising temperature on single thalli of *H* *. banksii* and assemblages dominated by a canopy of *H* *. banksii* , a sub-canopy of *Cystophora* *torulosa* and a basal turf of *Corallina* *officinalis* (hereafter referred to as ‘assemblages’). Due to the larger biomass of full assemblages, larger incubation chambers were required to accommodate them. However, the chambers were built of the same materials, including the same thickness of the lid. The same incubation protocol was used for single thalli and assemblages.

Single thalli of *H* *. banksii* were incubated in tubular Perspex chambers of internal 15 cm diameter (8 mm thick) and 15 cm height with flat 10 mm Perspex lids. These were kept at a constant temperature using a water jacket, consisting of a second Perspex tube surrounding the incubation chamber. The light source was above the incubation chambers and directed through the lid and not the sides of the chambers. Water was pumped from a temperature-controlled water bath to the cooling jacket of each chamber using a submerged magnetic pump. The internal temperature of incubation chambers was verified using internal temperature loggers. To prevent boundary layers forming on the algal surface, which could potentially limit photosynthesis, chambers were constantly mixed using a 3 cm magnetic stirrer (at 500 rpm). Water samples were extracted with a 1 mL syringe inserted through a tap in the lid and oxygen concentrations were measured in a Clark-type oxygen electrode (Strath Kelvin Microcell MC100) and cross-referenced with a Hach LDO probe (Model HQ40d).

The full algal assemblages were incubated in large chambers. These were 8 mm thick clear Perspex tube (30 cm high, 25 cm diameter), with a 10 mm thick clear Perspex base plate and lid [23]. Temperature was controlled by placing chambers in a constant temperature water bath, which was altered to experimental temperatures (verified using internal temperature loggers). The water within the incubation chambers was mixed using a submerged magnetic water pump that circulated the seawater in a turbulent vortex motion. Because of the size of the assemblages, the water in the chambers was exchanged after incubations at two irradiance levels to ensure that saturation did not occur and no nutrients became limiting. To prevent distortion of algal NPP due to respiration of invertebrates, all visible invertebrates were removed before incubations by freshwater dipping and physical removal.

Algae were incubated under various light intensities using a Phillips Discharge metal halide lamp limited to photosynthetically active radiation (PAR) wavelengths, with irradiance adjusted using neutral density filters to give five levels of irradiance (150, 300, 600, 1500, and 2000 μmol m^–2^ s^-1^). NPP was measured as changes in oxygen concentration using a Hach LDO meter (Model HQ40d). Dark respiration was obtained by covering the chamber to omit light. Measurements of dark respiration were performed at least 30 minutes after algae had been exposed to light. In total, six replicate assemblages were incubated under each light intensity and temperature combination. Following incubations, algae were dried for 24 h in a conventional oven at 50 °C and dry weights were recorded. NPP, GPP and respiration were standardized by dry weight of algae and/or area of the substratum.

### Analysis of photosynthetic parameters

Several photosynthetic characteristics were calculated in the laboratory and field incubations using photosynthesis vs. irradiance curves (*P-E*) across temperatures. These were: α, the light-use efficiency of the *P-E* curve; *R*, the respiration rate of the assemblage; *E*_*c*_, the compensation point of the assemblage; and *P*_*m*_, the NPP at saturating irradiance. Photosynthesis curves of single assemblage components were fitted against irradiance (i.e., *P-E* curves) using the exponential relationship described by Walsby [37]:

$$Pc=Pm[1-\exp(\frac{-\alpha E}{Pm})]+R$$

Where *P*_*m*_ is the maximum photosynthetic rate at light saturating irradiances, *E* is irradiance level, *R* is the rate of respiratory oxygen consumption (g O_2_ gDW^-1^ h^-1^), α is the light-use efficiency at light-limiting irradiances. The light-use efficiency (*α*) was calculated as the slope of a linear regression between 0-150 μmol m^-2^ s^-1^ for each replicate assemblage. Differences in photosynthetic parameters were tested using ANOVA. Also, differences in respiration rate (*R*), compensating irradiance (*E_c_*) and the light-use efficiency (*α*) were compared between single thalli and assemblages using ANCOVA.

Q_10_ values were calculated as the change in rate (GPP and respiration) over a 10°C temperature change. For laboratory assemblages the Q_10_ was calculated between 10-20°C and 15-25°C, and for field assemblages it was calculated between approximately 10-20°C. Q_10_ values were calculated for each replicate assemblage and averaged.

## Results

### Impacts of *in situ* temperature variation on assemblages and components

**Figure 1:**
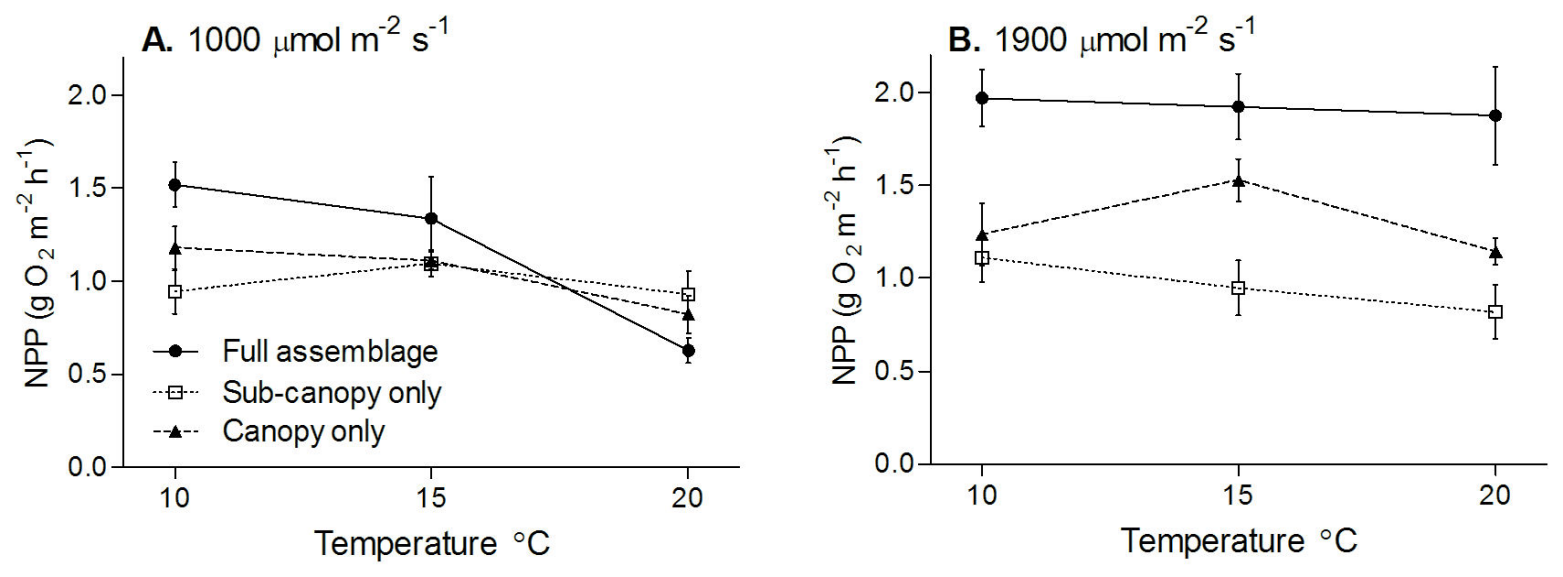
Effects of temperature on NPP of intact *in situ* macroalgal assemblages dominated by a canopy of *H. banksii*, the sub-canopy only and the canopy only (mean ±SE). NPP is shown at moderate irradiance, binned between 900-1100 μmol m^-2^ s^-1^ (A) and high irradiance, binned between 1800-2000 μmol m^-2^ s^-1^ (B).

*In situ* *H* *. banksii* -dominated assemblages had elevated respiration rates with increasing temperature (Table 1), but there was almost no difference in *P*_*m*_ or α, most likely caused by seasonal temperature and irradiance acclimation processes (Figure 1). However, there was a trend of increasing irradiance required to reach compensation with increasing temperature (*E*_*c*_; F_2,9_ = 13.0, P = 0.0066).

**Table 1:** Photosynthetic parameters of *in* *situ* *H. banksii*-dominated assemblages (±SE) at three temperature ranges.

**Temperature**	*R* (mg O2 gDW^-1^ h^-1^)	*P*_*m*_ (mg O2 gDW^-1^ h^-1^)	*α*	*E*_*c*_ (μmol m^–2^ s^-1^)
**8-12°C**	0.12 (0.02)	0.57 (0.04)	0.006 (0.0005)	48.39 (2.5)
**13-17°C**	0.16 (0.02)	0.6 (0.02)	0.006 (0.0012)	57.17 (2.8)
**18-22°C**	0.24 (0.03)	0.58 (0.05)	0.006 (0.0008)	76.26 (5.7)
**ANOVA**				
**F_2,9_**	9.5	0.35	0.05	13
**p**	0.014	ns	ns	0.0066

Parameters were; assemblage respiration (*R*), maximum NPP (*P*_*m*_), light-use efficiency (*α*), and irradiance at compensation (*E*_*c*_). Data were standardized by dry weight of algal material for comparison with laboratory data. ANOVA were used to test differences in photosynthetic parameters between temperature ranges.

NPP of *in situ* assemblages and the isolated community components (canopy only and sub-canopy only) had varied responses to temperature at moderate light intensities (1000 µmol m^-2^ s^-1^; Figure 1A). The canopy alone showed declining NPP with rising temperature, whereas the sub-canopy had its greatest average NPP at 15°C. Intact assemblages had lower NPP at 20°C than either the canopy alone or the sub-canopy alone. At 1000 µmol m^-2^ s^-1^, there was a significant interaction between temperature and canopy treatments, driven by the large decline in NPP of the full assemblage at 20°C (canopy treatment x temperature, F_4,18_ = 3.0, p = 0.048). At high light intensities (1900 µmol m^-2^ s^-1^) NPP of the sub-canopy alone declined with rising temperature, whereas full assemblages and the canopy alone had no overall trend with rising temperature (Figure 1B). The canopy treatment had an effect on NPP at 1900 µmol m^-2^ s^-1^ (canopy treatment, F_2,18_ = 27.8, p < 0.0001), but there was no significant effect of temperature and no interaction. Increasing respiration rates and compensating irradiance could have drastic consequences for the balance of primary productivity and respiration, but due to the seasonal variation in temperature, these effects cannot be corroborated without controlled laboratory experiments.

Species composition varied across seasons, but the dominant canopy and sub-canopy components remained somewhat constant, with *H* *. banksii* , *C* *. torulsa* and *C* *. officinalis* present in all plots during all seasons (Figure S1). The canopy of *H* *. banksii* had an average cover over 85% across all seasons, with the basal layer of *C* *. officinalis* contributing over 30% and the sub-canopy fucoid *C* *. torulosa* contributing over 15% during each season. A range of ephemeral macroalgae was found in the sub-canopy throughout the year, but the total number of species changed little (mean 7.8 ± 0.4 SE). Ephemeral species such as *Colpomenia* *sinuosa* , *Champia* *novae* *-zealandiae* and *Lophothamnionhirtum* were present at different times during the year, but rarely contributed more than 5% to overall cover.

### Effects of rising temperature — assemblages and single thalli

Community composition of macroalgal assemblages differed slightly among replicates in terms of species richness (average species richness of 8 ±SE 0.3) and percent cover of macroalgae (Table 2). However, *Hormosira* *banksii* , *Cystophora* *torulosa* , *Carpophyllummaschalocarpum* and *Corallina* *officinalis* were always present and although species richness of the sub-canopy varied among replicates, all replicate assemblages had between 7 and 9 species in total and between 5 and 7 sub-canopy species (average sub-canopy species richness 6 ±SE 0.2). The canopy of *Hormosira* *banksii* made up, on average, 45.3% (±SE 6.3) of the total biomass and the basal layer of *Corallina* *officinalis* made up 21.2% (±SE 3.8) of total biomass, while all of the combined sub-canopy species contributed 33.5% (±SE 2.7) of the total biomass.

**Table 2:** Average percent cover, average dry weight (±SE) and the canopy position of macroalgal species found in the assemblages used for laboratory incubations.

**Species**	**Ave % cover (SE**)	**Dry Weight g (SE**)	**Canopy position**
*Hormosira* *banksii*	88.25 (7.6)	70.6 (20.4)	Canopy
*Corallina* *officinalis*	38.25 (5.3)	33.1 (13.3)	Basal
*Cystophora* *torulosa*	16.5 (5.4)	15.2 (4.4)	Sub-canopy
*Carpophylummaschalocarpum*	4.75 (4.3)	15.8 (6.2)	Sub-canopy
*Champianovouzealandia*	8.75 (4.9)	9.1 (4.6)	Sub-canopy
*Adenocystisutricularis*	3.3 (3.5)	2.4 (0.3)	Sub-canopy
*Lophothamnionhirtum*	3.125 (1.3)	4.2 (2.2)	Sub-canopy
*Colpomenia* *sinuosa*	3.08 (0.3)	2.08 (0.4)	Sub-canopy
*Ulva* *spp*	2 (0.3)	1.4 (0.3)	Sub-canopy
*Janiamicrathrodia*	1 (0.4)	2.0 (0.4)	Sub-canopy

**Figure 2:**
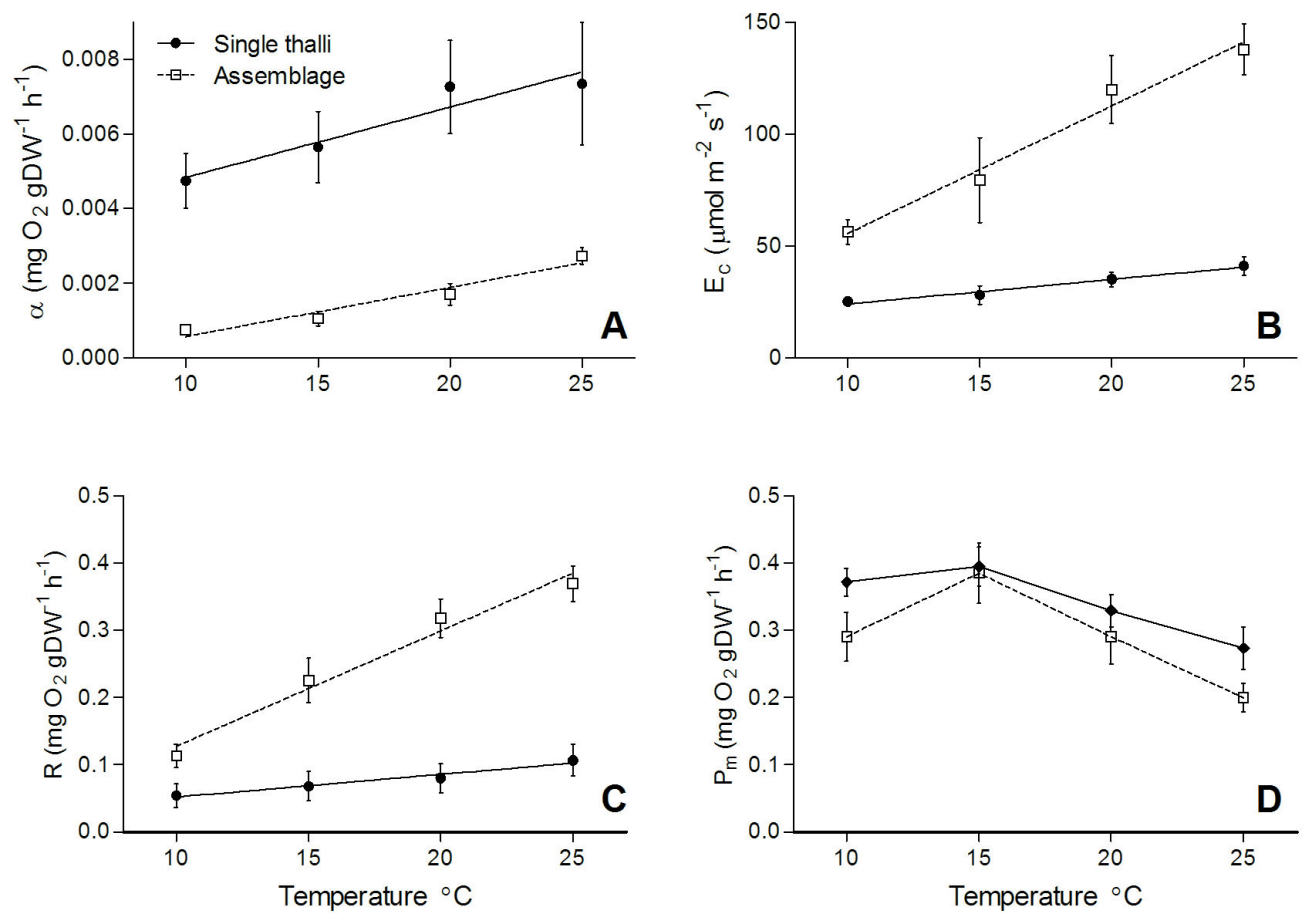
Effects of temperature on four photosynthetic parameters of *H. banksii* alone (single thalli) and *H. banksii* dominated assemblages (mean ±SE) in laboratory experiments. Parameters are the light-use efficiency or α (A), compensation point or *E*c (B), respiration or *R* (C) and maximum NPP or *P*_*m*_ (D) of *P-E* models.

Laboratory-based experiments showed that increasing temperature caused a shift in several photosynthetic parameters of assemblages dominated by a canopy of *Hormosira* *banksii* . Temperatures rising between 10-25°C resulted in an increase in light-use efficiency (α; Figure 2 A; r^2^ = 0.75, F_1,14_ = 42.4, p < 0.0001) and irradiance at the compensation point (*E*_*c*_; Figure 2 B; r^2^ = 0.64, F_1,15_ = 24.4, p = 0.0002). Respiration changed linearly with temperature (Figure 2 C; assemblage r^2^ = 0.87, F_1,14_ = 102.2, p < 0.0001), with Q_10_ values of 2.5 and 3.2 between 10-20 °C and 15-25 °C respectively (Table 3). Q_10_ of GPP was lower in the higher temperature range at all light intensities, and in the highest light treatment Q_10_ was below 1, which indicated a decline in GPP with rising temperatures. Although *in situ* assemblages showed no change in α or *P*_*m*_, controlling for acclamatory processes using acute laboratory experiments showed that some of the laboratory vs. *in situ* differences may be related to seasonal acclimation to light and temperature.

**Table 3:** Average Q_10_ of full assemblages dominated by *H. banksii.*

**Q_10_**		**Temperature range**	
	**Irradiance (µmol m^-2^ s^-1^**)	10-20°C	15-25°C
**GPP**	**300**	2.8	2.2
	**600**	2.1	1.4
	**1500**	1.3	1
	**2000**	1.2	0.9
**Respiration**	**0**	2.5	3.2

Q10 of GPP at four light intensities and respiration were calculated across two temperature ranges 10-20°C and 15-25°C.

A comparison of photosynthetic parameters of single thalli and assemblages showed that assemblages responded more negatively to rising temperatures than did single *H* *. banksii* thalli. Light-use efficiency (*α*) increased with rising temperatures (temperature: F_1,23_ = 42.4, p < 0.0001), but to a similar degree in both treatments (Figure 2A; treatment x temperature interaction: F_1,45_ = 1.47, p = 0.29). However, light-use efficiency values were much higher for single thalli than they were for assemblages (treatment: F_1,45_ = 36.1, P < 0.001). Both the increasing respiration rates with increasing temperature (temperature x treatment interaction; F_1,45_ = 47.2, p < 0.001) and the increase in the compensation irradiance (*E*_*c*_) with increasing temperature (temperature x treatment interaction; F_1,45_ = 36.1, P < 0.001) were more pronounced for assemblages than for single thalli (Figure 2B, C). Maximum NPP (*P*_*m*_) increased between 10 and 15°C both for assemblages and single thalli, but declined with increasing temperature. Although there was a greater decline in average NPP in the assemblages, there was no significant interaction of treatment x temperature (F_1,45_ = 0. 0008, p = 0.97), but NPP was lower for assemblages than for single thalli (treatment: F_1,45_ = 4.6, p = 0.037).

**Figure 3:**
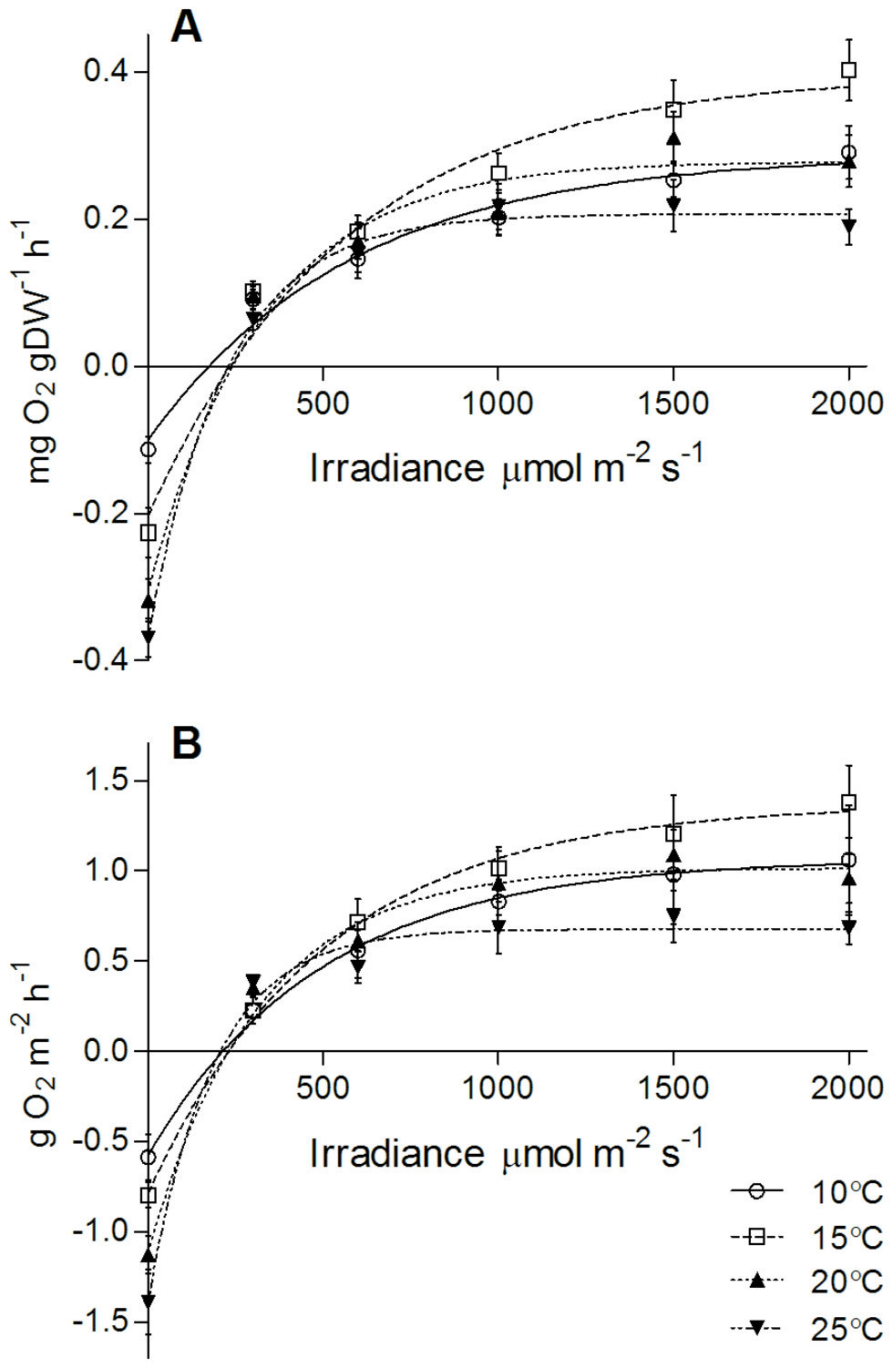
Response of NPP (±SE) in the laboratory to rising temperatures in macroalgal assemblages dominated by *H. banksii* at four treatment temperatures 10, 15, 20 and 25°C. Curves for each temperature are fitted with rectangular hyperbole. Data are standardized by (A) dry weight of algal material (mg O_2_ gDW^-1^ h^-1^) and (B) area of substrate (g O_2_ m^-2^ h^-1^).

The *P-E* curves generated for assemblages dominated by a *H* *. banksii* canopy in laboratory experiments had a marked response to rising temperatures (Figure 3 A and B). A change of 5°C (10-15°C) caused a rise in NPP across the light gradient, but increasing thermal stress (20°C and above) caused a decline in NPP, particularly at high light intensities. Although both methods of standardization (area or biomass) had similar responses to irradiance and temperature, variation was greater when standardized by area. *P*_*m*_ was significantly different between *P-E* curves at each temperature (F_3,131_ = 11.6, p < 0.0001) as was light use efficiency (α; F_3,131_ = 4.6, p = 0.0043). In the two curves centered on 10 and 15°C there was no sign of saturation of photosynthesis, whereas temperatures centered on 20 and 25°C caused saturation of photosynthesis, and possibly photoinhibition at high irradiance.

## Discussion

The acute effects of temperature change appear to have had an overall negative impact on the net primary productivity (NPP) of full assemblages through a large increase in respiration. Furthermore, laboratory-based experiments showed that at high irradiance and rising temperatures there was increasing photoinhibition, as seen in other macroalgae [29,30] and in coral zooxanthellae symbionts [38]. In contrast to laboratory results, however, natural *in situ* assemblages showed no signs of an enhanced photoinhibition in response to the combination of high light and temperature. These differences between lab and field studies may be related to the photo-acclimation of *in situ* assemblages over seasonal scales, which can cause differences in photosynthetic efficiency associated with seasonal variation in pigment content [39]. These results reflect the need to understand how natural communities will respond to climate change using approaches which account for natural variation in both environmental variables and community composition.

While we acknowledge that *in situ* assemblage NPP over an annual cycle may reflect seasonal variation in community structure and pigment content, the response of real communities to temperature across its natural range gives insight into how they may be affected by anthropogenic change. Loss of canopy-forming species worldwide has shifted many communities into being dominated by turfs [40,41] and understanding how multiple stressors impact these fucoid-dominated assemblages has significant implications for NPP and ecosystem functioning [16,17,42]. Furthermore, mechanistic approaches analysing multiple stressors have the potential to improve levels of confidence in predicted range shifts of species [43].

One of the few studies examining temperature effects on layered assemblages showed rises in both gross primary productivity (GPP) and respiration rates with increasing temperature [26]. Our results indicated a greater rise in the Q_10_ of respiration than of GPP, as has been observed in other autotrophs [12,27,31,44]. It has also been shown that some microphytobenthic communities can regulate the Q_10_ throughout the year, with higher values in winter (Q_10_ = 3) and lower in summer (Q_10_ = 1.2 [45]); our results indicated a similar range of Q_10_ values for GPP (Q_10_ = 1.1-2.5). The mismatch in rates of respiration and GPP could have far-reaching consequences to ecosystem NPP [24,46,47] because any potential enhancement of GPP with temperature can be drawn down by increased respiration rates, thereby affecting NPP. Furthermore, our results show that the shift in *P-E* curves with rising temperature could result in a significant decline in NPP during periods of elevated irradiance. These interactive effects suggest that even moderate rises in sea-surface temperature could cause a shift in community composition. For example, some invasive species have been shown to be more tolerant of pH and thermal stress than similar native species [26].

It is possible, and even probable, that the mechanisms operating at the assemblage level may be quite different from those operating at the species or thallus level. For example, temperature can have very different effects on NPP at saturating and non-saturating irradiance levels [10]. The impacts of low light and high temperature have been observed in macroalgae [10] and seagrasses [11], showing that autotrophs growing in low light conditions have lower optimum temperatures for photosynthesis. This has implications for assemblage NPP, given that species can be exposed to both high and low irradiance, depending on their positions in the canopy and tidal height [19]. Although canopies may be experiencing enhanced GPP due to Q_10_ effects of temperature on enzymatic processes, it is possible that the sub-canopy may be experiencing negative effects from temperature because increasing compensation points with rising temperature could force sub-canopy components into a state of net respiration.

Our experiments on responses to rising temperature of single thalli and assemblages showed that full assemblages are more prone to elevated respiration, but may be better able to mitigate photoinhibitory effects at high irradiance [15]. However, the acute response to the highest temperature indicated that assemblages are affected by the combined stresses of high irradiance and temperature, an effect commonly observed in coral zooxanthellae symbionts, which show a dramatic decline in photosynthetic rate [48]. We also found that at moderate light intensities, *in situ* assemblages were more affected by rising temperatures than the canopy or sub-canopy components alone, but at high light intensities *in situ*, intact assemblages were unaffected by temperature. Although it was not possible to relate this response directly to higher respiration of the sub-canopy, which was subjected to a low light environment, our data showed that at moderate above-canopy irradiance, the sub-canopy irradiance was often below the compensating irradiance for the dominant basal species *Corallina* *officinalis* [15]. As rising temperature shifts the compensating irradiance upwards, sub-canopy species may be less able to cope with rising temperatures in such light environments and will therefore exhibit greater respiration rates at higher temperatures.

Increasing temperatures may affect macroalgal assemblages in three ways: 1) higher respiration rates can change the ratio of photosynthesis to respiration, decreasing long-term net primary productivity; 2) the combination of high temperature and high irradiance may increase photoinhibition, thereby decreasing NPP during periods of high irradiance [38]; and 3) canopy layering may cause increased respiration in full assemblages because algae beneath a canopy experience, on average, lower light intensities and the negative effects of temperature will be exacerbated. Although increasing temperature had a minimal effect on respiration rates of single thalli tested in isolation, the variable delivery of light to lower canopy layers and enhanced rates of respiration may have much larger negative impacts on total assemblage NPP than predicted by single thalli responses.

## Supporting Information

Figure S1 **Percent cover (SE) of macroalgal species at Wairepo reef, Kaikoura New Zealand between October 2009 till July 2010.**
(TIF)
