# Supplementary figures and images for: Impacts of Temperature on Primary Productivity and Respiration in Naturally Structured Macroalgal Assemblages
Source: PLoS One. 2013 Sep 13;8(9):e74413. doi: 10.1371/journal.pone.0074413 (PMC3772813; doi:10.1371/journal.pone.0074413)

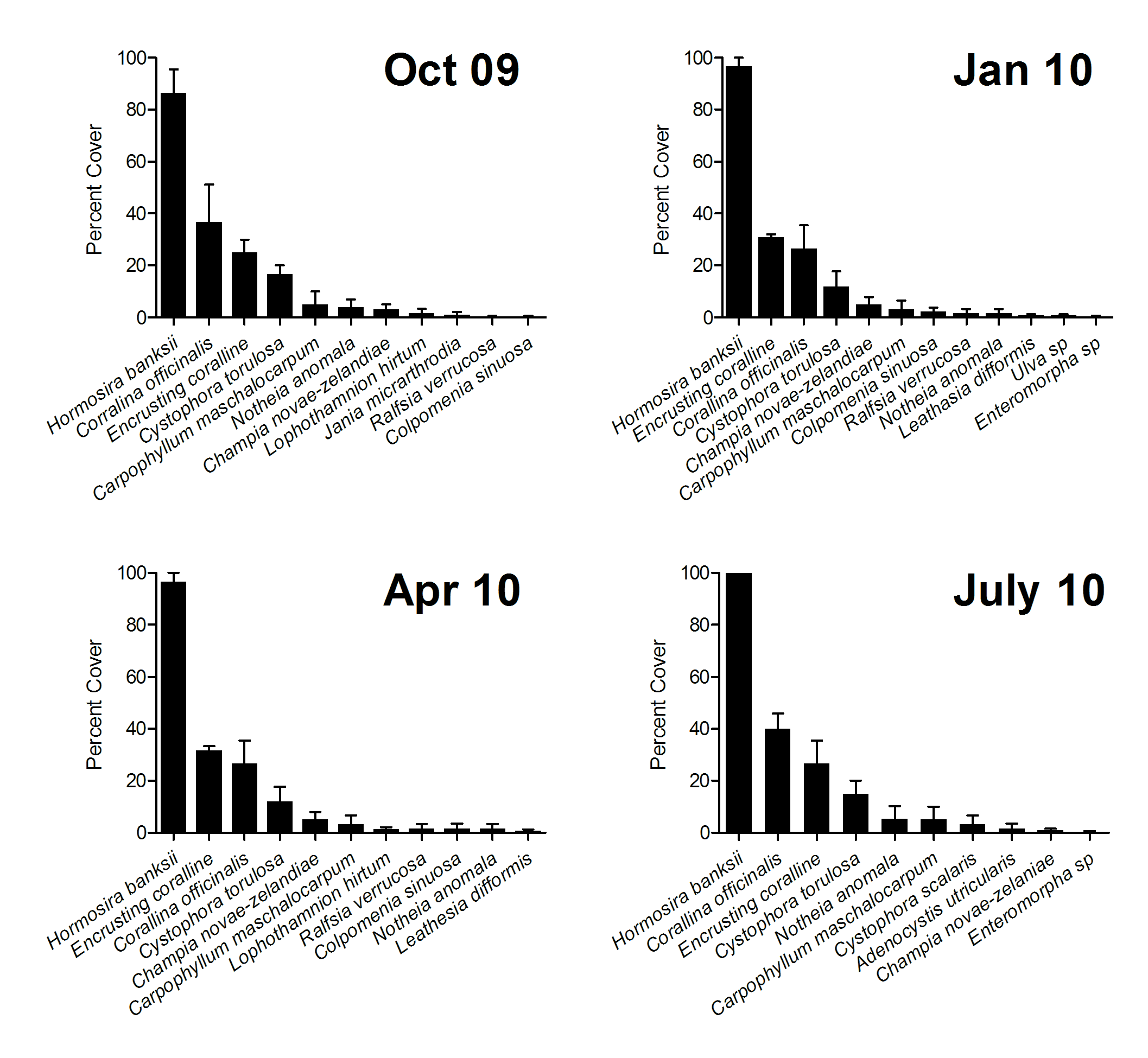

Supplement: Figure S1 — Percent cover (SE) of macroalgal species at Wairepo reef, Kaikoura New Zealand between October 2009 till July 2010. (TIF) [file pone.0074413.s001.tif]
